# A test of multimodal communication in humans using 881 judgements of men and women's physical, vocal, and olfactory attractiveness

**DOI:** 10.1016/j.heliyon.2023.e16895

**Published:** 2023-06-08

**Authors:** Megan Nicole Williams, Coren Lee Apicella

**Affiliations:** a University of Pennsylvania, Solomon Laboratories, 3720 Walnut Street, Philadelphia, PA, 09104, USA; b Division of Psychology, Department of Clinical Neuroscience, Karolinska Institutet, Stockholm, Sweden

**Keywords:** Multimodal communication, Multisensory communication, Attractiveness, Olfaction, Body odor, Multiple messages hypothesis, Redundancy hypothesis

## Abstract

Human mate value is assessed on numerous variables including, reproductive potential and disease resistance. Many of these variables have been correlated with judgments of physical, vocal, and odor attractiveness. While some researchers posit that attractiveness judgments made across different sensory modalities reflect the same underlying variable(s) (i.e., the information is redundant), others suggest that judgments made in different modalities reflect different variables. Previous studies of human attractiveness indicate that attractiveness judgments of others’ faces, bodies, and voices are intercorrelated, which is suggested to support the redundancy hypothesis. Less is known about body odor attractiveness. Only one study has simultaneously investigated the relationships between judgments of body odor, face, and voice attractiveness finding weak positive associations, but small effect sizes. In this study, we empirically investigate the correlation between different modalities of attractiveness in men and women in the largest sample to date (N = 881 ratings). For men, we find no correlations between modalities of attractiveness. However, for women we find odor, face, and voice attractiveness are weakly correlated. Moreover, a general attractiveness factor (i.e., a common underlying variable) modestly contributed to the observed correlations between modality-specific attractiveness judgments, providing some evidence for the redundancy hypothesis.

## Highlights

• Participants rated others' odor, face, and voice attractiveness, N = 881 ratings.
• For men, there were no correlations between modalities of attractiveness.
• For women, all modalities of attractiveness were weakly correlated.
• A common variable modestly contributed to women's multimodal attractiveness.
• Findings provide limited evidence for both the redundancy and multiple messages hypotheses.

## 1 Introduction

Person perception often entails rapid and spontaneous judgments, including judgments of attractiveness [e.g., [1]]. Attractive individuals fare better on diverse measures of economic, health, and social outcomes [2,3]. Predictably, a voluminous research literature has considered the factors underlying attractiveness judgments, with most attention paid to visual inputs (e.g., faces) and, to a lesser extent, auditory inputs (i.e., voices). Even less work has considered the role of odor in attractiveness judgments, in part because humans have been inaccurately labeled as poor smellers [[4], [5], [6], [7]]. However, most consequential social interactions, including those involving courtship and mating, usually occur in-person where information about individuals can be readily extracted from how a person looks, sounds, and smells. Yet, few studies have examined these inputs simultaneously, despite scientific consensus that person perception [e.g., [[8], [9], [10]]], and specifically attractiveness judgements [for review see, [11]], involve multisensory inputs. Such data is important for assessing the extent to which each of these sensory modalities provide unique, rather than redundant information.

Attractiveness preferences are thought to be evolved species-typical adaptations for choosing high quality mates [12]; that is, mates who are able to successfully interact with their social and physical environments to acquire resources and reproduce [[13], [14], [15]]. Human attractiveness judgments are multimodal, such that they involve more than one sensory input [for review see, [11]]. The reason for this multimodality is debated, with arguments stemming from research on the evolution of animal signaling and sexual selection for multiple ornaments. One hypothesis, the redundancy (or ‘back up’) hypothesis, states that multiple traits provide redundant or overlapping information about mate quality [16]. Redundancy can work to amplify messages, thus making them more detectable and memorable [for review see, [17]]. Redundancy is also beneficial in situations where messages are unreliable and communication channels are prone to disruption. Having multiple channels through which to communicate information helps to ensure the message is received [for reviews see, [17,18]]. Moreover, redundancy can reduce dishonesty in signaling because only individuals of high quality have the resources necessary to produce multiple traits communicating the same information [16,[18], [19], [20]]. In contrast, the multiple messages hypothesis states that each trait broadcasts unique information regarding an individual's mate quality [[18], [19], [20], [21]]. Multiple messages may be evaluated together and indicate an individual's overall mate quality, or different receivers may attend to different components in accordance with their own condition and genetic makeup [for review see, [19]]. To assess whether redundant or unique information is being conveyed by different traits, human attractiveness researchers have typically started by examining 1) whether multiple sensory modalities independently affect global assessments of attractiveness and, 2) correlations between attractiveness judgments in different modalities.

Relatively few studies have compared the contributions of different sensory modalities to overall (i.e., global) judgments of attractiveness. In fact, this literature has largely been limited to how different visual inputs contribute to overall judgements of physical attractiveness (i.e., perceptions of faces and bodies). And studies suggest that both faces and bodies [e.g., [[22], [23], [24]]] independently contribute to overall perceptions of attractiveness. Less work has examined voices, but there is some evidence to suggest that voices independently contribute to global judgments of attractiveness as well [[9],e.g., [[25], [26], [27]]]. These findings are often interpreted as evidence of multiple messages (i.e., unique information is conveyed by each modality of attractiveness). We are unaware of research that has explored the contribution of body odor to global judgments of attractiveness.

More commonly, researchers measure how attractiveness judgments across modalities correlate. Numerous studies report that women's facial and vocal attractiveness positively correlate, with the strength of the association ranging from weak [r = 0.2; ,[27]] to medium [r = 0.5; ,[28]] [8,[27], [28], [29], [30], [31], [32], but see, 33], however the same relationship is generally not found in men [8,27,[31], [32], [33], but see, 34]. While few studies have compared judgments of body odor attractiveness with other modalities of attractiveness, there is some evidence that judgments of body odor and facial attractiveness as well as body odor and vocal attractiveness are positively correlated [[35], [36], [37]]. For example, a recent meta-analysis of published and unpublished studies found weak positive associations between ratings of body odors and faces (*r* = 0.1, *k* = 25), and between body odors and voices (*r* = 0.1, *k* = 9), and also observed no sex differences in the magnitude of these effects [38]. Additionally, a recent study that used a naturalistic speed dating setup to assess the relationship between pre-date multimodal (i.e., visual, auditory, and olfactory) attractiveness ratings and post-date decisions to meet again, found that only visual attractiveness ratings significantly predicted post-date decisions. Moreover, their results revealed similar weak positive correlations between pre-date static ratings of attractiveness in all modalities for men and women, however the correlations between auditory and olfactory attractiveness ratings were not as robust. Further, all reported effect sizes were small, suggesting correlations between modalities of attractiveness were not strong [39].

In contrast to research demonstrating that sensory inputs independently contribute to measures of global attractiveness, concordance between judgments of attractiveness in different modalities is usually suggested to support the redundancy hypothesis [e.g., 40] because concordance is thought to indicate that a common trait, such as reproductive potential, is being communicated by each attractiveness modality. Yet, neither research paradigm (global assessments or correlational studies) fully distinguishes between the redundancy and multiple messages hypotheses because the information being conveyed by different sensory modalities could be both concordant and unique. For example, a woman's face may indicate that she is immunocompetent while her voice may indicate that she has high reproductive potential. Both messages separately indicate high mate quality and judgments of her facial and vocal attractiveness may be positively correlated, but not necessarily because they reflect a common underlying variable. Thus, the current study estimates the relationship between face, voice, and odor attractiveness and a latent general factor of attractiveness. Consequently, our analysis indicates how much a common trait contributes to the observed correlations between modality-specific attractiveness judgments allowing us to better distinguish between the redundancy and multiple messages hypotheses.

Here, we simultaneously investigate the relationship between judgments of body odor, face, and voice attractiveness by having groups of men and women rate the attractiveness of opposite-sex participants independently in each modality (i.e., face, odor, and voice). If judgments of attractiveness do not covary between modalities, then this may suggest that each modality conveys unique information (i.e., multiple messages). On the other hand, evidence of concordance between modalities of attractiveness could indicate that overlapping (i.e., redundant) information is being conveyed. However, in contrast to previous research that has only investigated correlations between modalities, we also estimate the relationship between each modality of attractiveness and a latent general factor of attractiveness. If we find evidence of concordance between ratings of odor, face, and voice attractiveness, then this analysis will help us to differentiate between the strength of evidence for both the redundancy and multiple messages hypotheses.

## 2 Materials and methods

### 2.1 Participants

We recruited *n* = 102 men and *n* = 96 women from a large urban university using a web-based subject pool (SONA system) to take part in this study. Participants received course credit for participation. The study entailed 2 laboratory sessions that took place on different days. In the first session, participants were called “donors” because they provided an odor and voice sample, and had their photograph taken. In the second session, participants were called “raters” because they rated the attractiveness of a subset of opposite-sex participants’ body odors, voices, and photographs. Participation occurred in same-sex groups of 1–9 participants (*Med.* = 4). An average of 5 study sessions were conducted 3 days per week from October 14, 2019 to December 9, 2019. There were 22 different second sessions with all male participants and 21 different seconds sessions with all female participants. Across rating sessions, the number of opposite-sex face, voice, and body odor stimuli rated varied. Face, voice, and odor stimuli from the same donor were used within a rating session, however different rating sessions used face, voice, and odor stimuli from different donors. Thus, stimuli from some donors were rated more, and others were rated less (range: 2–15 ratings per stimuli). In total, 1284 opposite-sex attractiveness ratings were made.

This study investigated attractiveness ratings of the opposite sex. Therefore, participants that reported “no” to identifying as heterosexual or chose not to identify their sexuality were excluded from all analyses (*n* = 16). Moreover, an additional 9 participants were excluded from analyses for failing to comply with fragrance guidelines (see section 2.2 *Donor compliance*). Thus, observations from 85 men (mean age = 20.39, *SD* = 3.54) and 88 women (mean age = 19.63, SD = 1.13) were used when forming the final estimation sample (see section 2.5 *Statistical Analyses*). 61.2% of men identified as White, 8.2% Black, 20.0% Asian or Pacific Islander, and 9.4% Hispanic, whereas 42.5% of women identified as White, 13.8% Black, 31.0% Asian or Pacific Islander, and 9.2% Hispanic. In addition, 36.9% of men reported being in a romantic relationship while 28.4% of women reported being in a romantic relationship.

Informed consent was obtained for each participant before both study sessions. The Office of Regulatory Affairs at the University of Pennsylvania approved all experimental procedures (IRB Protocol #834085).

### 2.2 Donor compliance

Two days prior to body odor collection, donors were given a series of dietary, behavioral, and hygiene restrictions. These restrictions are standard procedure for limiting contamination of “natural” body odor [e.g., 41,42]. Moreover, the day of body odor collection, donors were asked to refrain from (1) showering, (2) using deodorant, and (3) using any fragranced products, such as lotions and perfumes. A compliance survey was administered before body odor sampling, and t-tests were conducted comparing raters’ intensity and attractiveness judgments for donors that complied or failed to comply with compliance measures. Compliance was generally high. However, we found that fragrance use led to significantly higher odor attractiveness ratings for both men and women, meaning that men and women wearing fragrances or deodorant were judged to smell more attractive. Other compliance measures did not significantly affect odor attractiveness and intensity ratings when disaggregated by sex, which is how results are reported. Thus, we excluded only those donors that failed to comply with fragrance restrictions from further analyses. However, it should be noted that when male and female ratings were pooled, noncompliance with behavior and shower guidelines were also associated with significantly higher odor attractiveness ratings (see Supplementary Tables 1 and 2).

### 2.3 Rater screening

Several variables are thought to affect olfactory performance, including smoking, nasal congestion, smell loss, and medication usage. During the second study session, we asked raters to complete a screening survey pertaining to these variables prior to making any odor ratings. We conducted t-tests comparing odor attractiveness and intensity ratings between raters (overall and disaggregated by sex) that met or failed to meet (i.e., reported smoking, nasal congestion, smell loss, and/or medication use) screening criteria. We found no statistically significant differences between attractiveness or intensity ratings based on these screening variables (see Supplementary Tables 3 and 4); therefore, we felt justified including raters regardless of their responses on the screening survey.

### 2.4 Procedures

#### 2.4.1 Odor collection

Participants visited the lab twice, on separate days, first as donors and then as raters. Both visits were approximately 75 min. Body odor was collected on 4x4-inch sterile absorbent compresses (Johnson & Johnson) that were secured to participants’ axillae with skin safe (Nexcare) tape for 1 h. Compresses were frozen at −20° Celsius and thawed once, 30 min prior to use in rating sessions.

##### 2.4.1.1 Photographing

An instant camera (Fujifilm Instax Mini 70; 60 mm f/12.7 lens; automatic exposure control) was used to take photographs (62 mm × 42 mm) of donor faces. Donors were asked to remove makeup, eyewear, and jewelry, and to pull long hair back. Donors were photographed from a standardized distance (4.5 ft) in a black cotton t-shirt with a neutral expression while sitting against a light gray background. The camera was mounted on a tripod and the tripod height was adjusted for each donor so that the camera was level with the donor's face. Only a donor's face, neck, and collar of the provided black t-shirt were captured in an image.

##### 2.4.1.2 Voice recording

Donor voices were recorded while reading the “Rainbow Passage” [43] into a cardioid, omnidirectional condenser microphone (Logitech for creators, *Blue* Snowball; 16-bit/48 kHz) at a distance of approximately 5 inches. Recordings were made using the application software, Audacity. We uploaded a 7-s Mp3 file of each recording while donors said “When the sunlight strikes raindrops in the air, they act as a prism and form a rainbow” into a private SoundCloud account.

##### 2.4.1.3 Attractiveness ratings

During the second sessions, an odor rating station, a photograph rating station, and a voice rating station were setup, and each was managed by an experimenter. Samples (i.e., body odor, photographs, and voice recordings) from the same donors were placed at the appropriate rating stations 30 min before a rating session began. Raters made independent odor, voice, and face ratings. Each donor sample had a unique code so that raters could not match samples from the same donor across rating stations. Raters were randomly assigned an order to filter through rating stations with only a single rater at a rating station at a time.

Odor samples were contained in wide-mouth, amber, polypropylene jars. Jars contained compresses from both axillae of a single donor. An experimenter, wearing odorless cotton gloves, removed lids from jars one at a time and held the jars while a rater smelled the headspace. The order that odor samples were presented was randomized for each rater. After the rater smelled the jar's headspace, the experimenter replaced the jar's lid while the rater made a series of judgments about the sample's odor. Raters were asked to rate the intensity of the odor on a 7-point Likert scale, with 7 representing very intense. Then, raters made four judgments related to attractiveness (i.e., how pleasant, how attractive, how sexy, how much they liked …) using 7-point Likert scales, with 7 representing the highest score (e.g., very attractive). The scores for each of the four judgments were summed to create an overall attractiveness score (see Supplementary Table 5).^1^

Photographs of donor faces were presented by an experimenter in a randomized order for each rater. Raters made four judgments using the same scale described for odor, but with the wording changed to reflect faces. Additionally, raters were asked, “Do you know the person in this photograph?”. Observations where a rater knew the donor were excluded from all (i.e., face, odor, and voice) analyses.

Voice recordings were played by an experimenter in a randomized order for each rater. Raters wore large, over-the-ear, closed-back, noise-cancelling headphones (JVC stereo headphones, HA- G101) while at the voice rating station. Raters made four judgments using the same scale described for odor, but with the wording changed to reflect voices.

### 2.5 Statistical Analyses

#### 2.5.1 Estimation sample

In this section, we describe the construction of the core estimation sample used in our primary analyses of the sources of correlation between different modalities of attractiveness. We begin with an original data set in which each observation contains information about how a rater evaluated an opposite-sex donor's facial attractiveness, odor attractiveness and vocal attractiveness (N = 1284 observations). Since no rater was asked to evaluate the same donor more than once, each row in the data set contains information about a unique (ordered) donor-rater observation. Next, we excluded from our original sample 1) observations where either the donor or rater identified as non-heterosexual, 2) observations where the rater recognized the donor from the facial photograph, and 3) observations with missing or incomplete data about some basic rater and donor characteristics (including demographic characteristics and compliance measures). Applying these 3 exclusions left 949 total observations. In a final step, we dropped observations if the donor indicated that they did not comply with the request to abstain from the use of any fragranced products or deodorant on the day of body odor sampling. Excluding observations with donors that failed to comply with fragrance and deodorant restrictions left 881 observations, 437 of which corresponded to a female rater judging a male donor (and 444 where a male rater judged a female donor). Exclusion criteria were used across all modalities (odor, face, and voice); thus, there was an equal number of observations corresponding to the same donor-rater dyads in each modality of attractiveness. For each donor, we defined the rater's judgment in a modality by taking the sum of the four attractiveness questions about the modality in question. Supplementary Table 6 provides summary statistics for each of the three aggregated modality variables, both for the overall sample and by rater's sex.

##### 2.5.1.1 Two-factor model

The primary goal of our empirical analyses is to estimate the strength of the relationship between a person's attractiveness across three modalities – face, odor, and voice – and to explore the sources of these relationships. We fit a simple model which assumes attractiveness in modality *j* is determined as follows:

$$m_{j}=\mu_{j}+\beta_{j}A+u_{j}$$

where j∈{F,O,V} denotes facial, odor, and voice attractiveness, and A is a latent general attractiveness factor and $u_{j}$ is a mean-zero modality-specific disturbance that is assumed to satisfy, for any pair of modalities j≠k, $E\left[u_{j}u_{k}\right]$ and, for any individual modality, $E\left[u_{j}A\right]$. The assumption that $E\left[u_{j}u_{k}\right]$ for any j≠k is substantively important and means that the specific factors that influence one modality (vocal attractiveness, say) are unrelated to the specific factors that influence the other (facial attractiveness, say). Thus, any within-person correlation between attractiveness in modalities j and k arises through A. For example, if A and the three measured m are normalized to have mean zero and variance one, we have:

$$E\left[m_{j}m_{k}\right]=E\left[\left(\beta_{j}G+u_{j}\right)\left(\beta_{k}G+u_{k}\right)\right]$$

$$=E\left[\left(\beta_{j}{\beta_{k}G}^{2}+\beta_{j}Gu_{k}+{\beta_{k}u}_{j}G+u_{j}u_{k}\right)\right]$$

$$=\beta_{j}\beta_{k}E\left(G^{2}\right)+\beta_{j}E\left(Gu_{k}\right)+\beta_{k}E\left(u_{j}G\right)+E\left(u_{j}u_{k}\right)$$

$$=\beta_{j}\beta_{k}$$

**Fig. 1:**
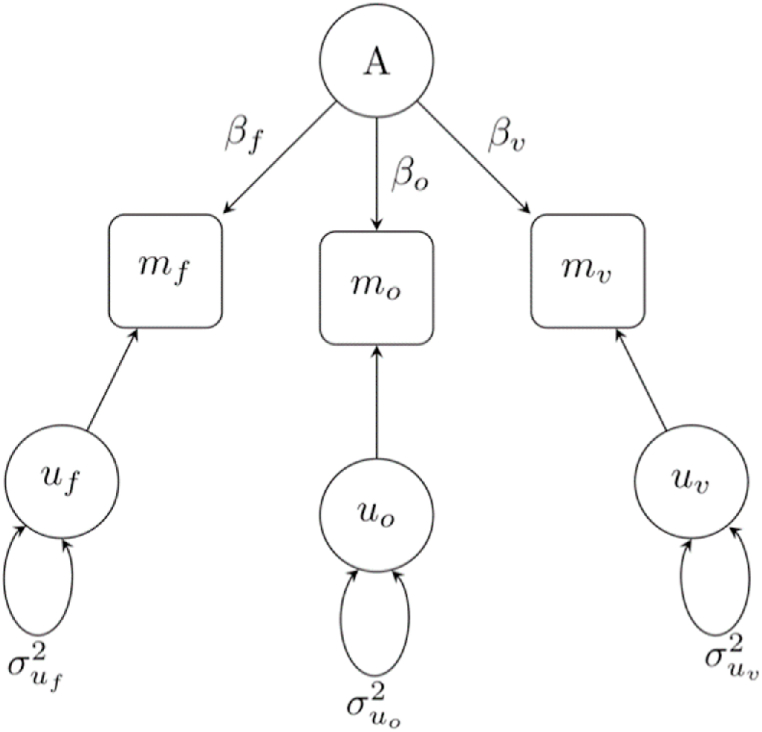
*Path Diagram Underlying Baseline Two-Factor Model of Attractiveness.* The figure displays a path diagram which assumes that attractiveness in a single modality (face, odor, or voice) is determined by a latent general attractiveness factor, A, and factors unique to a given modality (^*u*^). Modality specific factors influence one modality (odor attractiveness, say), and are unrelated to the factors influencing another (facial attractiveness, say). Therefore, any within-person correlation between attractiveness in different modalities arises through the latent general attractiveness factor, A.

Fig. 1 shows a path representation of our two-factor model. In this model, the variables $m=\left(m_{F},m_{O},m_{V}\right)$ are observed, with $m_{F}$ defined as the average of the four evaluations of the donor's facial attractiveness, and $m_{O}$ and $m_{V}$ defined analogously. Prior to model fitting, we residualize each of the attractiveness judgments on rater fixed-effects. We take this step to address concerns that within-person correlations in attractiveness judgments may otherwise reflect differences in how raters use the response scale (with some potentially being systematically more generous raters than others, in which case the correlations found could be spurious).

##### 2.5.1.2 Estimation

Prior to estimation, we normalize the latent variable A so its mean is zero and its variance is one. This normalization is not substantively important since it does not impact the standardized coefficient estimates numerically. That is, under any arbitrary rescaling of A, the standardized path coefficients we focus on in our discussion of the results are the same. We estimate the model parameters by maximum likelihood, clustering errors by the rater to take potential independence across observations into account. Since we do not demean or rescale any of the observed variables prior to estimation, the core of the model consists of nine parameters^2^:

• Three intercepts

$$\tilde{\mu }=\left({\tilde{\mu }}_{F},{\tilde{\mu }}_{o},{\tilde{\mu }}_{V}\right)$$

•Three disturbance variances

$${\tilde{\sigma }}_{u}^{2}=\left({\tilde{\sigma }}_{u,F}^{2},{\tilde{\sigma }}_{u,O}^{2},{\tilde{\sigma }}_{u,V}^{2}\right)$$

•Three unstandardized path coefficients, $\tilde{\beta }=\left({\tilde{\beta }}_{F},{\tilde{\beta }}_{O},{\tilde{\beta }}_{V}\right)$.

In practice, we follow the prior literature in estimating separate coefficients for male and female raters, doubling the number of parameters in the final model to 18. Where necessary, we use superscript s∈{m,f} to denote male and female coefficients, respectively. For some intuition on the identification, the model is just identified since we use 18 moments in the data to recover 18 parameters. For each sex, the nine moments are three means, three variances and three covariances. Procedurally, we estimate the model using the individual-level, untransformed, variables and then convert the unstandardized, original parameter estimates into standardized coefficients with more straightforward interpretations.

## 3 Results

Table 1 reports the correlation matrices for the residualized variables, both for the overall sample and separately by the sex of the rater. Supplementary Table 7 reports the same for the original variable.

**Table 1:** Correlations between odor, face, and voice attractiveness for all raters and disaggregated by sex, adjusted for rater fixed effects.

*All Raters*	Voice	Odor
Face	0.081*	−0.001
Odor	0.022	
*Male Raters*		
Face	0.144**	0.079
Odor	0.133**	
*Female Raters*		
Face	0.020	−0.066
Odor	−0.064	

*p < 0.05, **p < 0.01, ***p < 0.001.

Supplementary Table 8 reports the maximum-likelihood estimates of the untransformed model parameters. The standardized estimates derived from these estimates are shown in Table 2. For female donors (male raters), we estimate that a one-standard deviation-unit (SD unit) increase in general attractiveness, A, increases (i) facial attractiveness $\left(m_{F}\right)$ by 0.29 SD units (SE = 0.13, P < 0.011), (ii) odor attractiveness $\left(m_{O}\right)$ by 0.27 SD units (SE = 0.13, P < 0.040), and (iii) vocal attractiveness $\left(m_{V}\right)$ by 0.49 SD units (SE = 0.19, P < 0.008). According to the point estimates, the common attractiveness factor A is thus estimated to explain ${\hat{\beta }}_{F}^{2}=0.29^{2}∼8.6\%$ of the variance in facial attractiveness, $\left(m_{F}\right)$, ${\hat{\beta }}_{O}^{2}=0.27^{2}∼7.3\%$ of the variance in odor attractiveness $\left(m_{O}\right)$, and ${\hat{\beta }}_{V}^{2}=0.49^{2}∼24.2\%$ of the variance in vocal attractiveness $\left(m_{V}\right)$. Since a joint test of the null hypothesis that the three standardized path coefficients are identical – $\beta_{F}^{m}=\beta_{O}^{m}=\beta_{V}^{m}$ – fails to reject $\left(\chi^{2}\left(2\right)=0.63,P=0.730\right)\text{,}$ these findings do not provide strong evidence that the general attractiveness factor A is a better predictor of attractiveness in some modalities than others. But overall, our results provide evidence consistent with the hypothesis that a general attractiveness factor A contributes modestly to the observed correlations between modality-specific attractiveness judgments male raters make of female donors.

**Table 2:** Standardized coefficients from two-factor model.

	Male Raters (*N* = 444)				Female Raters (*N* = 437)			
	Estimate	SE	*P*	$R^{2}\left(\beta_{j}^{2}\right)$	Estimate	SE	*P*	$R^{2}\left(\beta_{j}^{2}\right)$
Standardized Path Coefs								
Face $\beta_{F}$	0.293	0.115	0.011	8.6%	0.145	0.264	0.584	2.1%
Odor $\beta_{o}$	0.270	0.131	0.040	7.3%	−0.457	0.885	0.606	20.9%
Voice $\beta_{v}$	0.492	0.186	0.008	24.2%	0.141	0.276	0.610	2.0%

For male donors (female raters), our results are qualitatively different and generally less informative. We estimate that in this group, a one-standard deviation-unit (SD unit) increase in general attractiveness, A, (i) increases facial attractiveness $\left(m_{F}\right)$ by 0.14 SD units (SE = 0.26, P = 0.584), (ii) *decreases* odor attractiveness $\left(m_{O}\right)$ by −0.46 SD units (SE = 0.89, P < 0.606), and (iii) increases vocal attractiveness $\left(m_{V}\right)$ by 0.14 SD units (SE = 0.28, P < 0.610). We emphasize that due to the low precision of these estimates, they do not provide much evidence that the standardized path coefficients are heterogeneous across domains. A joint test of the null hypothesis that the three standardized path coefficients are identical – $\beta_{F}^{f}=\beta_{O}^{f}=\beta_{V}^{f}$ – again fails to reject $\left(\chi^{2}\left(2\right)=0.85,P=0.265\right)$.

Our findings that within-person correlations across modalities are larger when women are rated by men than vice versa is consistent with most previous literature, which finds that female attractiveness tends to be more highly correlated across modalities than male attractiveness. To examine if the male-female differences in parameters are statistically significant, we conducted several formal hypothesis tests, the results of which are shown in Supplementary Table 9. The upper panel reports from three tests, each of which examines if one of the standardized path coefficients (corresponding to face, odor, or voice) can be equated across the two groups. None of the three individual tests rejects the null of equal parameters across the group. In the bottom panel, we report analogous tests for unstandardized path coefficients. We again fail to reject the null in all three cases. The bottom panel shows that a joint test of the three individual hypotheses also fails to reject.

In summary, our first objective was to estimate the strength of the relationship between a donor's attractiveness across three modalities – face, odor, and voice. For female attractiveness, we find that all attractiveness modalities are weakly correlated. For men, on the other hand, we find no correlation between their face, odor, and voice attractiveness. We cannot reject the null hypothesis that the male and female estimates are statistically similar. Our final objective was to investigate potential sources of relationships between attractiveness modalities, to distinguish between the redundancy and multiple messages hypotheses. We find that a latent general attractiveness factor (i.e., common underlying variable) contributes modestly to the correlations between women's face, odor, and voice attractiveness.

## 4 Discussion

For most of human evolutionary history courtship and mating has occurred in-person, where social information about an individual can be obtained from how they look, sound, and smell. Thus, sensory perceptions of other's attractiveness likely evolved in the context of being experienced together. Yet, little work has investigated how independent multisensory attractiveness ratings are related [for review, 11]. While some studies have examined the contributions of face and body to overall attractiveness (e.g., Brown et al., 1986; Mueser et al., 1984; Peters et al., 2007), and others have correlated facial and vocal attractiveness [8,[27], [28], [29],^31^,^32^,^40^], less published work has considered body odor attractiveness. Here, we simultaneously investigated the relationships between judgments of body odor, face, and voice attractiveness by having men and women rate the attractiveness of opposite-sex participants' body odors, faces, and voices (881 ratings). For women, we observed weak correlations between all modalities of attractiveness. However, for men, we found no correlations between modalities of attractiveness. Our findings are consistent with most prior studies, which also report within-person attractiveness correlations across sensory modalities in women [8,[27], [28], [29],^31^,^32^,^35^,^36^,^40^], but not men [8,27,[31], [32], [33]]. In contrast, Roth et al. [39] found weak, but significantly positive correlations between both male and female facial, vocal, and olfactory attractiveness. However, the authors suggest that their effect sizes were very small and larger studies would be necessary to detect nuances between male and female judgments. In the current study, we more than doubled their number of participants, but still cannot reject the null hypothesis that there are no sex differences in our estimates because, for an unknown reason, women's ratings of men were less precise. Thus, there could be no relationships between the modalities of attractiveness tested when women rate men, or the relationship could be comparable to what is reported for men rating women. This imprecision could potentially indicate that for men, even our larger sample size was too small to detect relationships between face, odor, and voice attractiveness.

Additionally, we estimated the relationship between face, voice, and body odor attractiveness, and a latent general factor of attractiveness, to better discriminate between the redundancy [16] and multiple messages hypotheses of multimodal signaling [20,21]. This analysis indicated how much a common trait (i.e., redundant information) contributed to the observed correlations between modality-specific attractiveness judgments*.* Our results revealed that a latent general attractiveness factor (i.e., common underlying variable) modestly contributed to the observed correlations between modality-specific attractiveness judgments in women. The contribution of a general attractiveness factor to modality-specific attractiveness to women's face, voice, and odor attractiveness provides some support for the hypothesis that female attractiveness in different modalities reflects a common trait (i.e., is redundant). However, because male attractiveness across domains seemingly was not significantly correlated, their face, voice, and odor may reflect several unique traits (i.e., multiple messages).

Indeed, researchers have posited that levels of female reproductive hormones, an indicator of fecundity and reproductive status [44,45], underlie perceptions of women's modality-specific attractiveness [46], and evidence exists supporting hormonally influenced attractiveness in each modality we tested. For example, women with relatively high estrogen levels have more feminine faces, a quality judged to be attractive [47]. In addition, women's voices are judged to be most attractive near ovulation, when estrogen levels are high [e.g., 48]. Finally, women with relatively high levels of oestradiol emit body odor judged to be most attractive [49]. In contrast, men's attractiveness in different modalities may convey distinct information, such as developmental stability, absence of harmful mutations, health status, and testosterone levels. For instance, though heavily debated, perceptions of men's facial attractiveness could reflect health and immunity [e.g., 50,51, but see, 52,53], whereas their vocal attractiveness may relate to trait or context-dependent dominance [e.g., 54,55]. However, there is little research on the precise fitness correlates of attractiveness preferences. In this study, we are similarly limited because we too cannot determine the fitness correlates to attractiveness in the modalities tested. Thus, what variable(s) associated with mate value is conveyed by modality-specific attractiveness in both men and women remains uncertain [for review see, 56].

### 4.1 Study limitations

In the current study, we did not control for menstrual cycle effects and other context-dependent variables, such as perceivers own attractiveness [57], which have been suggested to affect facial attractiveness ratings. In the future, studies should determine whether such context-dependent variables differentially affect modality-specific attractiveness to determine how context manipulates correlations between body odor, face, and voice attractiveness judgments. In addition, our choice of vocal stimuli could have altered the strength of correlations between vocal and attractiveness ratings in other modalities. Zäske et al. [33] found that face and voice attractiveness were uncorrelated when naturalistic speech was used, but a small significantly positive correlation was discovered between facial and vocal attractiveness when simple vowels were used instead. We used naturalistic speech (i.e., the rainbow passage) for voice samples, which cannot control for speaker characteristics, including dialect and speech patterns. Therefore, vocal attractiveness ratings could, in part, reflect socio-cultural preferences instead of preferences for vocal features. Yet, it is important to note that social judgments are based on holistic exposure to other's voices. Thus, from a sexual selection perspective, isolated vowel sounds are unlikely to contribute to human mate choice. Finally, although our findings provide some evidence that, in women, modality-specific attractiveness conveys information about a common trait, we only report weak correlations between independent multisensory attractiveness judgments. Thus, our results also indicate female body odors, faces, and voices provide unique information. In fact, the redundancy and multiple messages hypotheses are likely not mutually exclusive as some information may overlap between modalities of attractiveness (e.g., there is evidence to suggest reproductive hormone levels are associated with face, odor, and voice attractiveness [e.g., 48,49,58]) and other information may be unique to a specific modality (e.g., HLA and body odor [for review see, 59]). Humans likely evolved multiple methods of communicating and perceiving information about mate value.

The next step to determine how modality-specific attractiveness conveys redundant and/or unique messages is to determine the fitness correlates for features judged to be attractive. In addition, future research should investigate whether an individual's attractiveness in different modalities reflects disparate or concordant information, and how disparate/concordant information affects overall judgments of attractiveness. Moreover, future studies should examine how sensory environment and context alters the importance of different sources of information. For example, perhaps vision and audition are most important for mate choice at a distance or during first encounters, but smell may become more important as the proximity between individuals decreases and mating becomes more likely.

Mate preferences are based on numerous, often interacting, indicators of mate value. Yet, multisensory inputs to mate choice have received little attention, particularly olfaction. More studies are necessary to understand the contribution of body odor to mate selection. The current study is a first step and adds to our knowledge of the relationships between body odor attractiveness and attractiveness in other sensory modalities.

## Funding

This research did not receive any specific grant from funding agencies in the public, commercial, or not-for-profit sectors.

## Author contribution statement

Megan Nicole Williams: Conceived and designed the experiments; Performed the experiments; Analyzed and interpreted the data; Wrote the paper.

Coren Lee Apicella: Conceived and designed the experiments; Wrote the paper.

## Data availability statement

Data will be made available on request.

## Declaration of competing interest

The current version of the manuscript was approved by all authors. The authors declare no conflict of interest.

## Footnotes

1 Using a correlation matrix, we present the relationships between each of the summed attractiveness ratings in each modality as well as the relationship between each rating and the total attractiveness score in each modality. Questions within a given modality were highly correlated, thus we felt justified in summing each to create an overall attractiveness score within each modality.

2 See Supplementary Material for robustness analysis and discussion modeling variables as ordinal as opposed to continuous.
