# Supplementary material for: A test of multimodal communication in humans using 881 judgements of men and women's physical, vocal, and olfactory attractiveness
Source: Heliyon. 2023 Jun 8;9(6):e16895. doi: 10.1016/j.heliyon.2023.e16895 (PMC10277517; doi:10.1016/j.heliyon.2023.e16895)
Supplement: Multimedia component 1 [file mmc1.docx]

**A test of multimodal communication in humans using 881 judgements of men and women's physical, vocal, and olfactory attractiveness**

Supplementary Materials

**Comparison of model using ordinal as opposed to continuous variables**

We examined how sensitive our conclusions are to the decision to treat the variables as continuous as opposed to ordinal. To do this, we formulated and estimated an alternative model that treats the variables as ordinal instead of continuous. We follow the common convention of assuming that the ordinal variable defines cutoffs of the distribution of some latent variable. Without loss of generality, assume the Likert categories are ordered monotonically so that category 1 denotes the least attractive option, category 2 denotes the second least attractive option, and so on up to category 7, which denotes the most attractive option. A common approach to analyzing these types of data is to work with a model which assumes the category chosen by a rater evaluating facial attractiveness dfi ∈ {1, 2, 3, 4, 5, 6, 7}, is related to the unobserved variable of interest, m ̃ fj, as follows


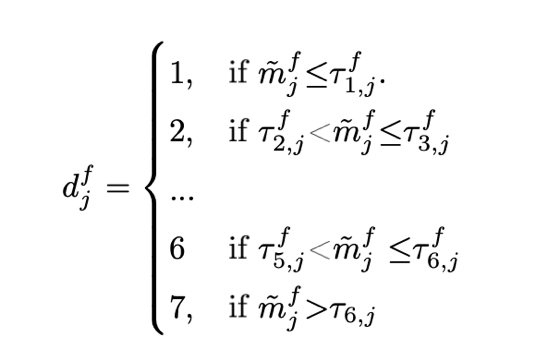


where j ∈ {1,2,3,4} denotes the specific item. The model we consider assumes each latent variable is normally distributed. We do not impose any constraints on the values of cutoffs/thresholds across items. The path diagram underlying the generalized structural equation model that considers the ordinal nature of the data is shown below.


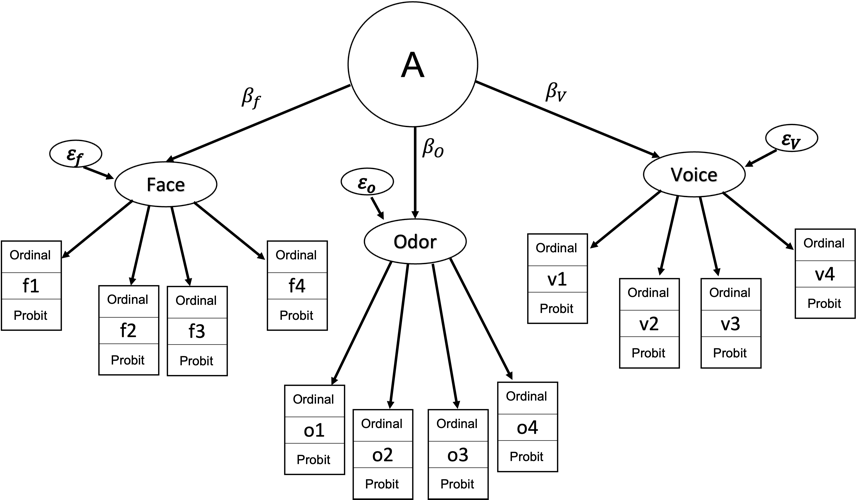


Remarks on Ordinal Model

We note that:

- The ordinal model can be estimated in a one-step procedure (even though doing so is computationally intensive). Ultimately, the coefficients of greatest substantive importance are the ones labelled βf , βm and βo in the path diagram. These allow us to estimate the proportion of variance in each latent factor explained by variance in A.
- The ordinal model has six cutoff parameters for each item-level measure. Since each of the three modalities is associated with four item-level measures, each with six cutoff parameters, the model has 24 × 3 = 72 cutoff/threshold parameters alone.
- Additional model parameters include a vector of standardized path coefficients βi = β1i , β2i , β3i , β4i , one for each of the modalities. Each vector can be interpreted as the estimated effects from a hypothetical multivariate regression of the latent modality variable on the four latent variables assumed to underly the four observed item-level measures. The vector is only identified up to a scalar transformation, so one of the coefficients (typically the first) is normalized to one.
- The model has a total of 96 parameters (or 192 if sex-specific parameters are estimated). By contrast, the model used in our main analyses has 9 parameters (18 if sex-specific parameters are estimated).

Comparison of Ordinal Model Estimates to Metric Model Estimates

We compared the estimated proportions of variance in each modality explained by A in our main analyses to analogous estimates from the generalized structural equation model described above, in which the data are treated as ordinal.

For example, the estimates shown here are for male raters evaluating female donors. We were reassured to see that the results are broadly similar. Specifically:

- The estimates from the metric (ordinal) model imply that the latent attractiveness factor A, explains 8.6% (9.2%) of the variance in Face.
- The estimates from the metric (ordinal) model imply that the latent attractiveness factor A, explains 7.3% (8.3%) of the variance in Odor.
- The estimates from the metric (ordinal) model imply that the latent attractiveness factor A, explains 24.2% (60.4%) of the variance in Voice.

**Supplementary Table 1**

*Comparisons of odor intensity ratings of samples from donors who complied (i.e., C) or did not comply (i.e., NC) with dietary, behavioral, fragrance, and shower guidelines. Results are presented in full and disaggregated by sex.*

|  |  | Diet | | Behavior | | Fragrance | | Shower | |
| --- | --- | --- | --- | --- | --- | --- | --- | --- | --- |
|  |  | C | NC | C | NC | C | NC | C | NC |
| Female Donors | |  | |  |  |  |  |  |  |
|  | Mean | 3.59 | 3.41 | 3.57 | 3.21 | 3.49 | 4.56 | 3.52 | 3.61 |
|  | SD | 0.83 | 0.94 | 0.81 | 1.42 | 0.84 | 0.53 | 0.87 | 0.54 |
|  | *N* | 62 | 22 | 78 | 6 | 80 | 4 | 78 | 4 |
|  | *t*-statistic | 0.84 | | 0.98 | | -2.53 | | -0.22 | |
|  | *p* | 0.40 | | 0.33 | | 0.01* | | 0.83 | |
| Male Donors | |  | |  |  |  |  |  |  |
|  | Mean | 3.88 | 3.95 | 3.97 | 3.35 | 3.86 | 4.31 | 3.90 | 3.85 |
|  | SD | 1.24 | 1.30 | 1.28 | 0.86 | 1.27 | 0.82 | 1.28 | 0.85 |
|  | *N* | 68 | 19 | 76 | 11 | 80 | 7 | 79 | 8 |
|  | *t*-statistic | -0.22 | | 1.55 | | -0.91 | | 0.10 | |
|  | *p* | 0.83 | | 0.12 | | 0.36 | | 0.92 | |
| All Donors | |  | |  |  |  |  |  |  |
|  | Mean | 3.74 | 3.66 | 3.77 | 3.30 | 3.67 | 4.40 | 3.71 | 3.77 |
|  | SD | 1.07 | 1.14 | 1.08 | 1.05 | 1.09 | 0.71 | 1.11 | 0.74 |
|  | *N* | 130 | 41 | 154 | 17 | 160 | 11 | 157 | 12 |
|  | *t*-statistic | 0.41 | | 1.69 | | -2.18 | | -0.19 | |
|  | *p* | 0.68 | | 0.09 | | 0.03* | | 0.85 | |
| *Note.* Raters made one intensity rating on a 7-point Likert scale; thus, intensity ratings range from 1 to 7.  * p < .05, **p < .01, ***p < .001  **Supplementary Table 2**  *Comparisons of odor attractiveness ratings of samples from donors who complied (i.e., C) or did not comply (i.e., NC) with dietary, behavioral, fragrance, and shower guidelines. Results are presented in full and disaggregated by sex.*   \|  \|  \| Diet \| \| Behavior \| \| Fragrance \| \| Shower \| \| \| --- \| --- \| --- \| --- \| --- \| --- \| --- \| --- \| --- \| --- \| \|  \|  \| C \| NC \| C \| NC \| C \| NC \| C \| NC \| \| Female Donors \| \|  \| \|  \|  \|  \|  \|  \|  \| \|  \| Mean \| 11.92 \| 11.80 \| 11.72 \| 14.16 \| 11.57 \| 18.28 \| 11.83 \| 14.74 \| \| SD \| 2.94 \| 3.29 \| 2.86 \| 4.29 \| 2.69 \| 1.93 \| 2.94 \| 2.66 \| \| *N* \| 62 \| 22 \| 78 \| 6 \| 80 \| 4 \| 78 \| 4 \| \| *t*-statistic \| 0.16 \| \| -1.94 \| \| -4.90 \| \| -1.94 \| \| \| *p* \| 0.87 \| \| 0.06 \| \| < 0.001*** \| \| 0.06 \| \| \| Male Donors \| \|  \|  \|  \|  \|  \|  \|  \|  \| \|  \| Mean \| 11.63 \| 12.24 \| 11.58 \| 13.08 \| 11.38 \| 16.18 \| 11.54 \| 13.97 \| \|  \| SD \| 3.77 \| 4.14 \| 3.86 \| 3.56 \| 3.71 \| 2.11 \| 3.82 \| 3.44 \| \|  \| *N* \| 68 \| 19 \| 76 \| 11 \| 80 \| 7 \| 79 \| 8 \| \|  \| *t*-statistic \| -0.60 \| \| -1.21 \| \| -3.36 \| \| -1.72 \| \| \|  \| *p* \| 0.55 \| \| 0.23 \| \| 0.001** \| \| 0.09 \| \| \| All Donors \| \|  \|  \|  \|  \|  \|  \|  \|  \| \|  \| Mean \| 11.77 \| 12.00 \| 11.65 \| 13.46 \| 11.48 \| 16.95 \| 11.69 \| 14.22 \| \|  \| SD \| 3.39 \| 3.67 \| 3.38 \| 3.74 \| 3.24 \| 2.22 \| 3.40 \| 3.10 \| \|  \| *N* \| 130 \| 41 \| 154 \| 17 \| 160 \| 11 \| 157 \| 12 \| \|  \| *t*-statistic \| -0.37 \| \| -2.07 \| \| -5.51 \| \| -2.50 \| \| \|  \| *p* \| 0.71 \| \| 0.04* \| \| <0.001*** \| \| 0.01* \| \|   *Note.* Raters made four attractiveness ratings on 7-point Likert scales. These ratings were summed to create an overall attractiveness score; thus, overall attractiveness ratings range from 4 to 28.  * p < .05, **p < .01, ***p < .001 | | | | | | | | | |

**Supplementary Table 3**

*Comparison of odor intensity ratings between raters who complied (i.e., C) or did not comply (i.e., NC) with smoking (i.e., nonsmoker), nasal congestion (i.e., no nasal congestion), lifetime smell loss (i.e., none), and medication usage (i.e., none) screening criteria. Results are presented in full and disaggregated by sex.*

|  |  | Smoker | | Nasal Congestion | | Smell Loss | | Medication | |
| --- | --- | --- | --- | --- | --- | --- | --- | --- | --- |
|  |  | C | NC | C | NC | C | NC | C | NC |
| Female Raters | |  | |  |  |  |  |  |  |
|  | Mean | 3.84 | 3.76 | 3.80 | 3.95 | 3.83 | 4.07 | 3.83 | 3.47 |
|  | SD | 1.04 | 0.80 | 1.05 | 0.88 | 1.02 | 1.29 | 1.04 | 0.19 |
|  | *N* | 78 | 5 | 72 | 11 | 80 | 3 | 83 | 2 |
|  | *t*-statistic | 0.17 | | -0.37 | | -0.39 | | 0.49 | |
|  | *p* | 0.86 | | 0.71 | | 0.70 | | 0.63 | |
| Male Raters | |  | |  |  |  |  |  |  |
|  | Mean | 3.56 | 3.70 | 3.54 | 3.68 | 3.55 | 3.76 | 3.56 | 3.69 |
|  | SD | 0.98 | 0.93 | 0.93 | 1.09 | 0.93 | 1.31 | 0.99 | 0.64 |
|  | *N* | 72 | 7 | 60 | 19 | 71 | 8 | 72 | 8 |
|  | *t*-statistic | -0.36 | | -0.58 | | -0.59 | | -0.36 | |
|  | *p* | 0.72 | | 0.57 | | 0.56 | | 0.72 | |
| All Raters | |  | |  |  |  |  |  |  |
|  | Mean | 3.71 | 3.73 | 3.69 | 3.78 | 3.69 | 3.85 | 3.70 | 3.64 |
|  | SD | 1.02 | 0.84 | 1.00 | 1.01 | 0.99 | 1.25 | 1.03 | 0.58 |
|  | *N* | 150 | 12 | 132 | 30 | 151 | 11 | 155 | 10 |
|  | *t*-statistic | 0.06 | | 0.43 | | -0.47 | | 0.18 | |
|  | *p* | 0.95 | | 0.67 | | 0.64 | | 0.86 | |
| *Note.* Raters made one intensity rating on a 7-point Likert scale; thus, intensity ratings range from 1 to 7.  * p < .05, **p < .01, ***p < .001 | | | | | | | | | |

**Supplementary Table 4**

*Comparison of odor attractiveness ratings between raters who complied (i.e., C) or did not comply (i.e., NC) with smoking (i.e., nonsmoker), nasal congestion (i.e., no nasal congestion), lifetime smell loss (i.e., none), and medication usage (i.e., none) screening criteria. Results are presented in full and disaggregated by sex.*

|  |  | Smoker | | Nasal Congestion | | Smell Loss | | Medication | |
| --- | --- | --- | --- | --- | --- | --- | --- | --- | --- |
|  |  | C | NC | C | NC | C | NC | C | NC |
| Female Raters | |  | |  |  |  |  |  |  |
|  | Mean | 11.89 | 10.93 | 11.71 | 12.61 | 11.88 | 10.67 | 11.72 | 13.97 |
|  | SD | 3.52 | 4.16 | 3.55 | 3.54 | 3.50 | 5.20 | 3.53 | 3.35 |
|  | *N* | 78 | 5 | 72 | 11 | 80 | 3 | 83 | 2 |
|  | *t*-statistic | 0.59 | | -0.78 | | 0.58 | | -0.89 | |
|  | *p* | 0.56 | | 0.44 | | 0.56 | | 0.38 | |
| Male Raters | |  |  |  |  |  |  |  |  |
|  | Mean | 11.79 | 11.28 | 11.65 | 12.08 | 11.70 | 12.22 | 11.69 | 12.02 |
|  | SD | 3.24 | 2.23 | 3.30 | 2.74 | 3.12 | 3.80 | 3.19 | 3.00 |
|  | *N* | 72 | 7 | 60 | 19 | 71 | 8 | 72 | 8 |
|  | *t*-statistic | 0.41 | | -0.52 | | -0.44 | | -0.28 | |
|  | *p* | 0.68 | | 0.61 | | 0.66 | | 0.78 | |
| All Raters | |  |  |  |  |  |  |  |  |
|  | Mean | 11.84 | 11.13 | 11.68 | 12.27 | 11.79 | 11.79 | 11.70 | 12.41 |
|  | SD | 3.38 | 3.01 | 3.42 | 3.01 | 3.31 | 4.00 | 3.36 | 2.96 |
|  | *N* | 150 | 12 | 132 | 30 | 151 | 11 | 155 | 10 |
|  | *t*-statistic | 0.71 | | -0.87 | | -0.00 | | -0.65 | |
|  | *p* | 0.48 | | 0.38 | | 1.00 | | 0.52 | |
| *Note.* Raters made four attractiveness ratings on 7-point Likert scales. These ratings were summed to create an overall attractiveness score; thus, overall attractiveness ratings range from 4 to 28.  * p < .05, **p < .01, ***p < .001 | | | | | | | | | |

| **Supplementary Table 5**  *Correlation matrices of attractiveness-rating-scale items.* | | | | | | |
| --- | --- | --- | --- | --- | --- | --- |
| Face  Face |  | Pleasant | Attractive | Sexy | Like |  |
|  | Pleasant |  |  |  |  |  |
|  | Attractive | 0.741 |  |  |  |  |
|  | Sexy | 0.651 | 0.878 |  |  |  |
|  | Like | 0.773 | 0.823 | 0.785 |  |  |
|  | Attractiveness Score | 0.868 | 0.944 | 0.909 | 0.927 |  |
| Odor | Pleasant |  |  |  |  |  |
|  | Attractive | 0.855 |  |  |  |  |
|  | Sexy | 0.768 | 0.855 |  |  |  |
|  | Like | 0.869 | 0.847 | 0.798 |  |  |
|  | Attractiveness Score | 0.934 | 0.951 | 0.915 | 0.940 |  |
| Voice | Pleasant |  |  |  |  |  |
|  | Attractive | 0.749 |  |  |  |  |
|  | Sexy | 0.643 | 0.853 |  |  |  |
|  | Like | 0.832 | 0.809 | 0.740 |  |  |
|  | Attractiveness Score | 0.886 | 0.937 | 0.889 | 0.929 |  |

**Supplementary Table 6**

*Summary statistics for face, odor, and voice attractiveness for overall sample and by rater’s sex.*

|  |  | All Ratings  (*N* = 881) | Male Ratings  (*N* = 444) | Female Ratings  (*N* = 437) |
| --- | --- | --- | --- | --- |
| Face | Mean | 12.633 | 13.124 | 12.135 |
|  | SD | 5.334 | 5.504 | 5.114 |
| Odor | Mean | 11.344 | 11.282 | 11.407 |
|  | SD | 5.383 | 4.963 | 5.783 |
| Voice | Mean | 15.070 | 15.126 | 15.014 |
|  | SD | 5.488 | 5.212 | 5.761 |

*Note.* Raters made four attractiveness ratings on 7-point Likert scales. These ratings were summed to create an overall attractiveness score; thus, a donor’s overall attractiveness score ranged from 4 to 28. The statistics above reflect the average attractiveness score within a given modality.

**Supplementary Table 7**

Correlations between odor, face, and voice attractiveness for all raters and disaggregated by sex.

| *All Raters* | Voice | Odor |
| --- | --- | --- |
| Face | 0.151*** | 0.082* |
| Odor | 0.093** |  |
| *Male Raters* |  |  |
| Face | 0.223*** | 0.110* |
| Odor | 0.197*** |  |
| *Female Raters* |  |  |
| Face | 0.081 | 0.059 |
| Odor | 0.012 |  |

* p < .05, **p < .01, ***p < .001

**Supplementary Table 8**

*Unstandardized coefficients from two-factor model.*

|  | Male Ratings (*N* = 444) | | | Female Ratings (*N*=437) | | |
| --- | --- | --- | --- | --- | --- | --- |
|  | Coef | SE | *P* | Coef | SE | *P* |
| Face |  |  |  |  |  |  |
| Path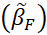 | 1.230 | 0.493 | 0.013 | 0.611 | 1.112 | 0.582 |
| Intercept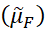 | 13.124 | 0.000 | 0.000 | 12.135 | 0.000 | 0.000 |
| Variance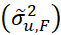 | 16.143 | 1.856 | 0.000 | 17.516 | 2.207 | 0.000 |
|  |  |  |  |  |  |  |
| Odor |  |  |  |  |  |  |
| Path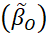 | 1.048 | 0.527 | 0.047 | -2.115 | 4.097 | 0.606 |
| Intercept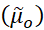 | 11.282 | 0.000 | 0.000 | 11.407 | 0.000 | 0.000 |
| Variance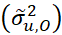 | 13.990 | 1.575 | 0.000 | 16.965 | 17.461 | 0.331 |
|  |  |  |  |  |  |  |
| Voice |  |  |  |  |  |  |
| Path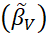 | 2.213 | 0.841 | 0.009 | 0.688 | 1.346 | 0.609 |
| Intercept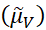 | 15.126 | 0.000 | 0.000 | 15.014 | 0.000 | 0.000 |
| Variance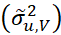 | 15.374 | 3.888 | 0.000 | 23.443 | 3.031 | 0.000 |
|  |  |  |  |  |  |  |
| log pseudolikelihood | *-*7640.416 |  |  |  |  |  |

**Supplementary Table 9**

*Tests of equal path coefficients across sexes.*

|  | | | | *Z* | *P>\|Z\|* |
| --- | --- | --- | --- | --- | --- |
| 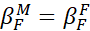Standardized Path Coefficients | | | |  |  |
| 1 |  |  |  | -0.51 | 0.607 |
| 2 | 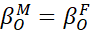 |  |  | -0.81 | 0.417 |
| 3 | 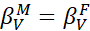 |  |  | -1.06 | 0.291 |
| Unstandardized Path Coefficients | | | |  |  |
|  |  |  | 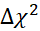 | 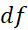 | 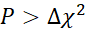 |
| 4 | 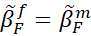 |  | 0.259 | 1 | 0.611 |
| 5 | 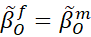 |  | 0.586 | 1 | 0.4439 |
| 6 | 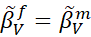 |  | 0.924 | 1 | 0.336 |
|  |  |  |  |  |  |
| Joint Test of (4)-(6) | | | 0.945 | 3.000 | 0.184 |
